# Ancient Mammalian and Plant DNA from Late Quaternary Stalagmite Layers at Solkota Cave, Georgia

**DOI:** 10.1038/s41598-019-43147-0

**Published:** 2019-04-29

**Authors:** M. C. Stahlschmidt, T. C. Collin, D. M. Fernandes, G. Bar-Oz, A. Belfer-Cohen, Z. Gao, N. Jakeli, Z. Matskevich, T. Meshveliani, J. K. Pritchard, F. McDermott, R. Pinhasi

**Affiliations:** 1 Department of Human Evolution, Max-Planck-Institute for Evolutionary Anthropology, Leipzig, Germany; 2 School of Archaeology, University College Dublin, Dublin, Ireland; 3 School of Medicine, University College Dublin, Dublin, Ireland; 4 Department of Evolutionary Anthropology, University of Vienna, Vienna, Austria; 5 CIAS, Department of Life Sciences, University of Coimbra, Coimbra, Portugal; 6 Zinman Institute of Archaeology, University of Haifa, Haifa, Israel; 7 Institute of Archaeology, The Hebrew University of Jerusalem, Jerusalem, Israel; 8 Department of Genetics, Stanford University, Stanford, USA; 9 Department of Prehistory, Georgian State Museum, Tbilisi, Georgia; 10 Israel Antiquities Authority, Jerusalem, Israel; 11 Departments of Biology, Stanford University, Stanford, USA; 12 Howard Hughes Medical Institute, Stanford University, Stanford, USA; 13 School of Earth Sciences, University College Dublin, Dublin, Ireland

**Keywords:** Genetics, Palaeoclimate, Archaeology

## Abstract

Metagenomic analysis is a highly promising technique in paleogenetic research that allows analysis of the complete genomic make-up of a sample. This technique has successfully been employed to archaeological sediments, but possible leaching of DNA through the sequence limits interpretation. We applied this technique to the analysis of ancient DNA (aDNA) from Late Quaternary stalagmites from two caves in Western Georgia, Melouri Cave and Solkota. Stalagmites form closed systems, limiting the effect of leaching, and can be securely dated with U-series. The analyses of the sequence data from the Melouri Cave stalagmite revealed potential contamination and low preservation of DNA. However, the two Solkota stalagmites preserved ancient DNA molecules of mammals (bear, roe deer, bats) and plants (chestnut, hazelnut, flax). The aDNA bearing layers from one of the two Solkota stalagmites were dated to between ~84 ka and ~56 ka BP by U-series. The second Solkota stalagmite contained excessive detrital clay obstructing U-series dating, but it also contained bear bones with a minimum age of ~50 BP uncalibrated years and ancient DNA molecules. The preservation of authentic ancient DNA molecules in Late Quaternary speleothems opens up a new paleogenetic archive for archaeological, paleontological and paleoenvironmental research.

## Introduction

Ancient DNA (aDNA) genomics is a valuable information source on past biological diversity and evolutionary trajectories of species^1–3^. A particular focus has been on the analysis of human bones yielding high coverage genomes of archaic humans^4–6^ and enabling novel insights into human dispersals and migrations^7–9^. Additionally, several studies employed a metagenomic approach to the study of DNA sequence data retrieved from soils and sediments from various environments, including caves^10^, lakes^11^, arid^12^ and arctic environments^13,14^. Slon *et al*.^15^ using a shotgun sequencing approach and analysing the deamination pattern for identification of authentic ancient DNA^16^, reported on the recovery of archaic human aDNA as well as other mammalian aDNA from archaeological deposits at several sites. This metagenomic research shows that not only bones but many other components of the archaeological and paleontological record, such as deposits themselves, may serve as a preservation medium for ancient DNA.

The retrieval of authentic aDNA strands from deposits is made possible by the binding of DNA to various sediment and soil components, including clays^17–19^, silica^20,21^, humic acids^22^ and calcite^23^. However, soil chemistry, e.g. pH^20^, and soil transformation processes, such as the dissolution and precipitation of minerals, greatly impacts preservation. Furthermore, post-depositional movement of sediment components through turbation, such as bioturbation, as well as other soil translocation processes, such as clay illuviation, may negatively impact the integrity and complicate the interpretation of aDNA found in sediments and soils^24,25^.

Speleothems are another potential source for aDNA and have long been explored as paleoenvironmental archives using other methods, mainly stable isotopes studies and U-series dating^26^. Paleoenvironmental studies most commonly use stalagmites, which form on the cave floor below a drip and in which calcite precipitates in distinctive and continuous layers. Preservation conditions for DNA are ideal inside the stalagmites and especially for those located deeper inside caves, where low temperatures limit the production of reactive oxygen species^27^, there is little exposure to UV light^28^ and a stable pH as well as very low permeability inside the stalagmite and hence a low risk of DNA migration between consecutive stalagmite layers. Few DNA studies have been conducted on speleothems and they are mainly restricted to the surface of speleothems^29–31^ with the exception of a study by Zepeda Mendoza *et al*.^32^, who analysed two samples from the inside of popcorn calcite from a dolerite granite gneiss cave. However, while they reported that aDNA was preserved inside the speleothems, they concluded that this type of speleothem is unsuitable as a biological paleoarchive^32^. ‘Popcorn’ calcite exhibits rather irregular and complex multi-dimensional growth patterns compared with the relatively simple sequential deposition of consecutive layers in stalagmites, making the latter a geometrically simpler and therefore more reliable archive.

**Figure 1:**
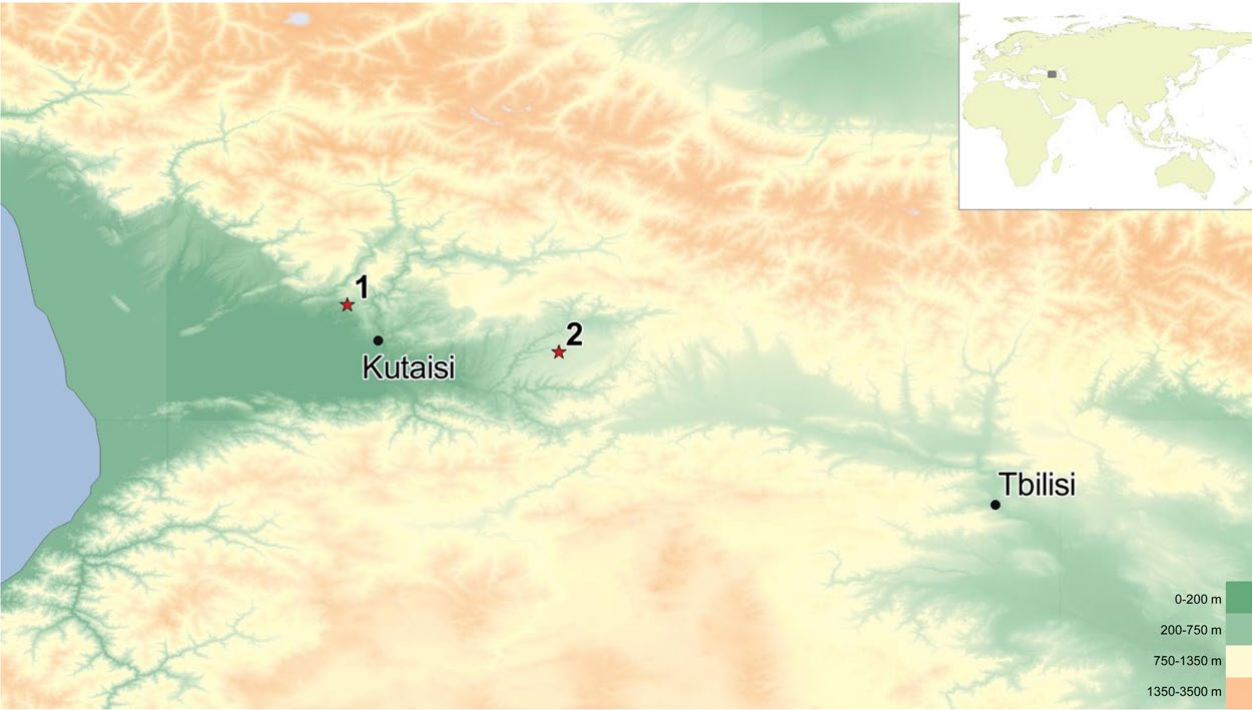
Location of the study sites. The studied cave sites are located in Western Georgia (map created with ASTER GDEM^59^): (1) Location of Satsurblia, Solkota, Melouri and Datvi Cave; (2) Location of Dzudzuana and Kotias Klde.

**Figure 2:**
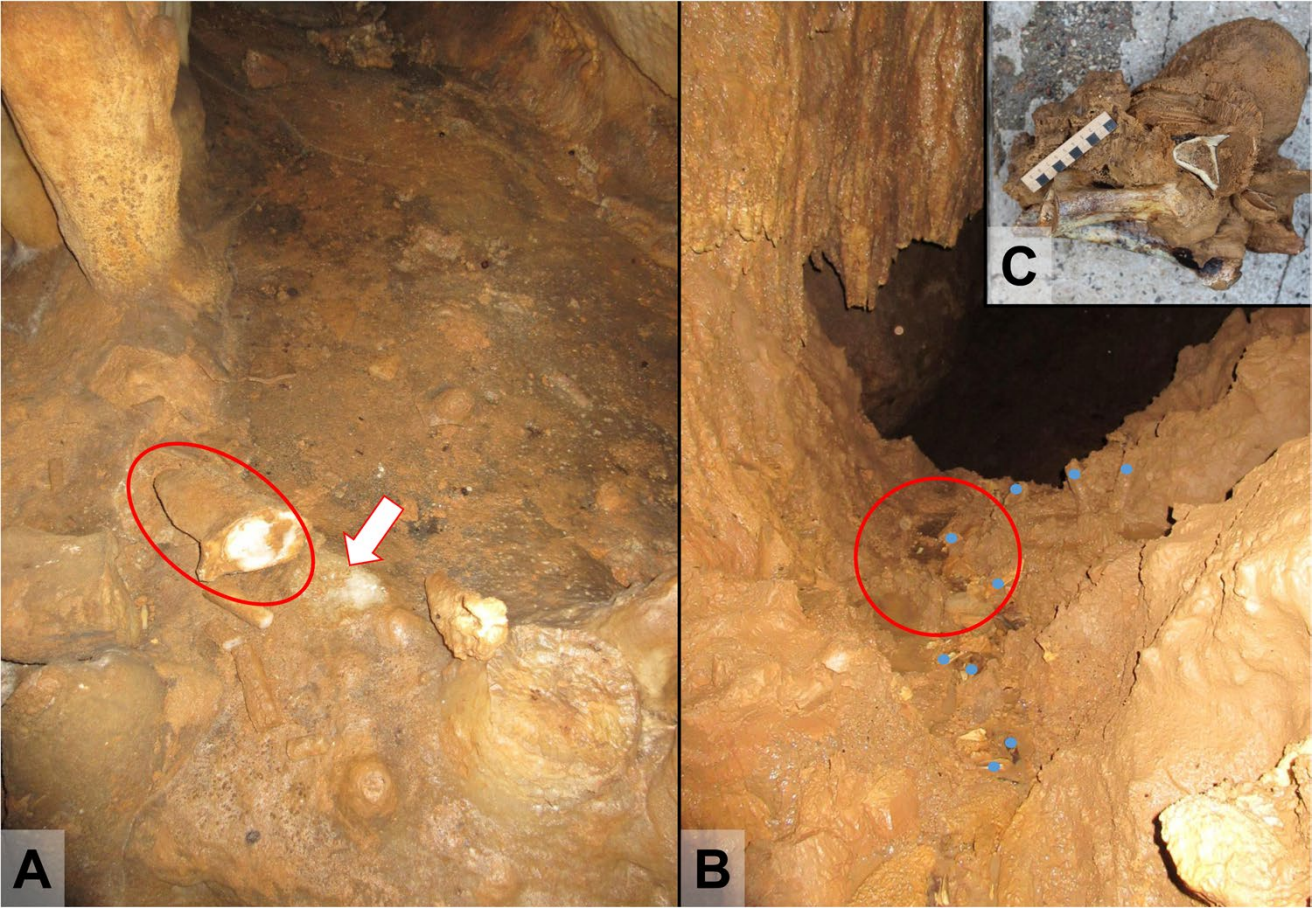
Sampling locations of the Solkota Cave stalagmites SKK 16 3 and 5 (photos taken by MCS). (**A**) The find spot of SKK 16 3 (red circle) next to its possible root (white arrow). Note the scarcity of sediment here. (**B**) Discovery location of SKK 16 5 (red circle) in a rill bed next to multiple bone remains (blue dots). (**C**) Stalagmite SKK 16 5 with cave bear bones at its base.

We here present a first metagenomic study exploring aDNA metagenomics combined with U-series dates of stalagmites from two caves from Western Georgia, Solkota and Melouri Cave, as archives on species that interacted with or inhabited these cave systems. In 2016, we surveyed six caves in the Imereti region of Georgia (Fig. 1): three archaeological cave sites - Satsurblia Cave^33^, Dzudzuana^34^, Kotias Klde^35^ - and three non-archaeological cave sites - Melouri Cave, Datvi Cave, Solkota. The latter three caves contained cave bear bones, but were not archaeologically explored, and only these sites had favourable speleothems for the aim of this study. Each of the three archaeological cave sites had a large entrance, permitting light and air to enter into the cave, which typically makes them less suitable for quantitative paleoenvironmental reconstructions based on stable isotope studies^26^. We therefore chose to proceed in our analysis with one stalagmite from Melouri Cave (MEL) and with two stalagmites from Solkota (SKK) (Fig. 2). Ancient DNA was detected in several locations inside the two Solkota stalagmites (SKK 16 3 and 5). However, analysis of the stalagmite from Melouri Cave revealed potential contamination and low preservation of DNA (see results below, SI Text 1 and Fig. 1) and we focus here on the Solkota samples.

Solkota cave lies near the village of Kumistavi above the river Semi. Solkota is part of the same karst system as Satsurblia Cave, Melouri Cave and Datvi Cave, the Tskaltubo karst system in the Sataplia-Tskaltubo Limestone Massif^36^. The cave entrance of Solkota is located in a sinkhole with a very steep slope and little light penetrating into the cave entrance. The rear of the cave consists of a steep, muddy slope leading upwards with bedrock exposed at the top of the slope. Another, former entrance may have been present here. Next to limestone boulders the cave contains clay-rich mud and water concentrated in ponds and rills. The cave is rich in speleothems (stalagmites, flowstone, stalactite, curtains, straws) as well as in bone and we also found three lithic flakes. Bones are often exposed in rill beds and we collected 40 bones. One bone was identified as capra, two as canids and the remaining 37 as cave bear (*Ursus spelaeus* or *Ursus deningeri*). We also observed several bear hibernation dens in the inner parts of the cave. We collected one large stalagmite, which had been growing on top of three cave bear long bones (SKK 16 5) from the secondary context of a rill bed (Fig. 2B,C). The bones comprised of right and left distal humerus and a distal shaft of a tibia. Carnivore gnaw marks were observed on the surface of the bones. We collected a second stalagmite (SKK 16 3) from the top of the slope at the rear of the cave, close to its potential root (Fig. 2A).

## Results

### U-series

**Figure 3:**
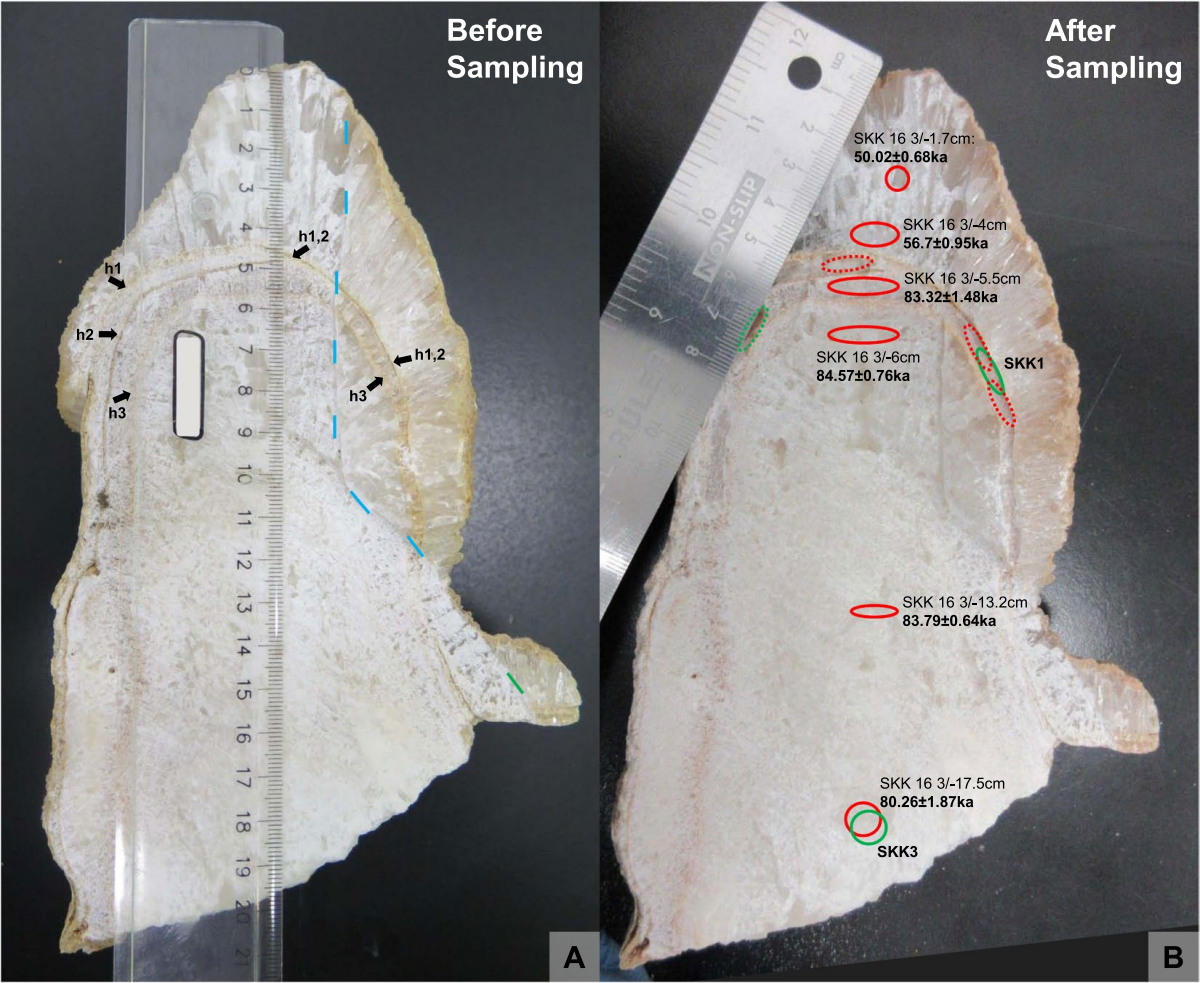
The cut stalagmite SKK 16 3 (photos taken by MCS). (**A**) SKK 16 3 before sampling. The stalagmite was partially cut open with a rock saw and then broken open (broken surface is to the right of the dashed blue line) to reduce contamination by the saw blade. Three dark lines stemming from hiatuses in speleothem formation can be observed at dfts of 4.5 (h1), 4.8 (h2) and 5.6 (h3) cms (black arrows). Note that hiatuses h1 and h2 combine to the right (h1, 2). (**B**) SKK 16 3 after sampling for DNA analysis and U-series dating. U-series samples (red, dotted line if unsuccessful analysis) and samples for DNA analysis (green, dotted line if unsuccessful analysis) were often taken in close association. Reliable U-series ages are reported next to their sampling location. Note however, that the age of 80.26 ± 1.87 ka in the same sampling locality as SKK3 is less reliable as it is out if stratigraphic order. DNA sample SKK1 was taken in the same layer as U-series sample SKK 16 3/−5.5, between hiatuses h3 and the combined hiatus h1 and h2 and dating to 83.32 ± 1.48 ka. Its age is capped by U-series ages from layers above (56.7 ± 0.95 ka) and below (84.57 ± 0.76). SKK1 may contain dust particles from the hiatus events.

**Figure 4:**
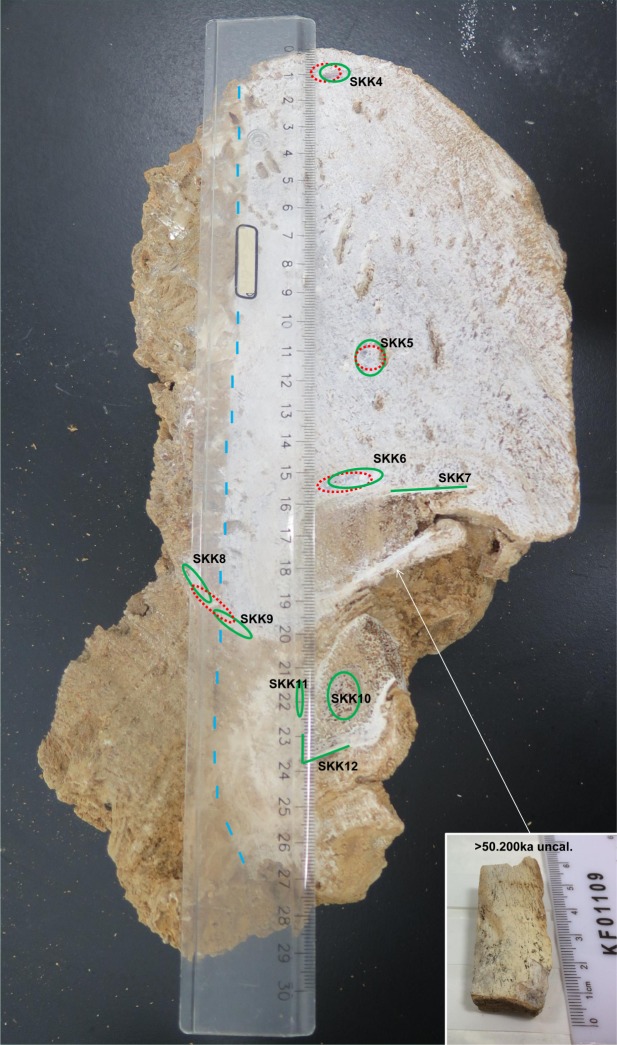
The cut stalagmite SKK 16 5 (photo taken by MCS). SKK 16 was sampled for U-series dating (red dotted line, unsuccessful analyses) and DNA analysis (green, dotted line if unsuccessful analysis), which include samples from the stalagmites as well as the incorporated bones (SKK 7 and 12 from cortical bone and SKK 10 from trabecular bone). Similar to SKK 16 3, stalagmite SKK 16 5 was also partially cut open with a rock saw and then broken open (left of the blue dashed line) and both contexts were sampled. Note the brown colour of the speleothem, indicating the presence of detrital clay, which impeded the U-series dating. However, each sample gave aDNA reads.

Uranium concentrations in speleothem SKK 16 3 are relatively low, typically in the range 35–70 ppb (Table 1). Three samples from SKK 16 3 (3/10, 3/6.5, 3/5) (Fig. 3) have high ^232^Th contents (c. 42–77 ppb) resulting in low (^230^Th/^232^Th) values (between 2.1 and 3.2), and therefore unacceptably large age uncertainties after corrections for detrital thorium have been applied (SI Table 1). Similarly, the ^232^Th contents for all samples from SKK 16 5 (Fig. 4) are too high to calculate ages for this speleothem (SI Table 1). However, six samples from SKK 16 3, corresponding to dft (depth from top) values of 17.5, 13.2, 6, 5.5, 4 and 1.7 cms, have low ^232^Th contents yield moderately high (^230^Th/^232^Th) ratios in the range 14.7–148 (Table 1), permitting the calculation of precise U-series ages following correction for the detrital clay component. As discussed in the Methods section, clay-rich samples from the cave were measured separately using a total dissolution approach to constrain the actual (^230^Th/^232^Th) value of the detrital component in the speleothems, considerably reducing the uncertainties in the corrected U-series ages compared to the standard approach of simply assuming a (^230^Th/^232^Th) value for the detrital component. Overall, detrital corrected U-series dates for the key stalagmite SKK 16 3 from Solkota range from 83.79 ± 0.64 ka at a depth from top (dft) of 13.2 cm to 50.02 ± 0.68 ka at a dft of 1.7 cm (Fig. 3). In detail however, considerable complexity in the speleothem’s growth history is evident.

**Table 1:** U-series data for speleothem SKK 16 3.

Sample	^238^U ppb	(^230^Th/^238^U)	(^234^U/^238^U)	(^230^Th/^232^Th)	^232^Th ppb	Age ka uncorrected	Age ka corrected
SKK16 3/17.5	37.083 ± 0.003	0.6317 ± 0.0020	1.1221 ± 0.0009	14.70 ± 0.04	4.8733 ± 0.0043	88.63 ± 0.54	80.26 ± ^1.87^_1.84_
SKK16 3/13.2	35.263 ± 0.004	0.6138 ± 0.0019	1.1245 ± 0.0014	148.10 ± 0.41	0.4466 ± 0.0030	84.59 ± 0.55	83.79 ± ^0.64^_0.63_
SKK16 3/6	35.34 ± 0.02	0.6140 ± 0.0017	1.1087 ± 0.0012	59.414 ± 0.153	1.1162 ± 0.0003	86.64 ± 0.50	84.57 ± ^0.76^_0.75_
SKK16 3/5.5	64.113 ± 0.005	0.6338 ± 0.0011	1.1156 ± 0.0010	19.086 ± 0.031	6.5056 ± 0.0012	89.95 ± 0.36	83.32 ± ^1.48^_1.46_
SKK 16 3/4	53.907 ± 0.003	0.5001 ± 0.0008	1.1546 ± 0.0007	17.157 ± 0.026	4.8015 ± 0.0012	61.07 ± 0.18	56.69 ± ^0.95^_0.94_
SKK16 3/1.7	38.183 ± 0.005	0.4510 ± 0.0009	1.1658 ± 0.0018	23.266 ± 0.047	2.2618 ± 0.0004	52.72 ± 0.24	50.02 ± ^0.68^_0.67_

Parentheses denote activity ratios. Dates reported in this table are considered reliable after detrital corrections have been applied (see SI Table 1 for dates strongly affected by detrital correction and with no reliable age calculation). The following decay constants were used: ^230^Th: 9.1577E-6, ^232^Th: 4.9475E-11, ^234^U: 2.826E-6, ^238^UE 1.551E-10. The final column on the right hand side shows the ages calculated after correction for detrital thorium using a measured (^230^Th/^232^Th) value of 0.95 ± 0.1 for the detrital end-member.

The date from the sample closest to the base of the speleothem (17.5 cm dft) yields an age of 80.26 ± 1.87 ka, out of stratigraphic order, and just outside the error limits of the next three dates above (83.79 ± 0.64, 84.57 ± 0.76, 83.32 ± 1.48 at dfts of 13.2, 6 and 5.5 cms respectively, Fig. 3). This may indicate some minor post-depositional migration of uranium in the lower section of the speleothem. Regardless, the similarity of the next three dates (all three within their 2σ errors) indicates an interval of very rapid speleothem growth around 84 ka and no detectable post-depositional uranium migration. Warm, wet intervals favour high speleothem growth rates and we note that this time interval coincides with climatic amelioration during Greenland Interstadial 21.1e (GI-21.1e)^37^ during Marine Isotope Stage (MIS) 5a.

Examination of the cut surface of stalagmite SKK16 3 reveals the presence of three distinctly visible depositional hiatuses at dfts of 4.5 (hiatus 1), 4.8 (hiatus 2) and 5.6 cms (hiatus 3) (Fig. 3). These provide clear evidence that the speleothem growth was discontinuous above the interval dated at 83.32 ± 1.48 ka. The DNA sample SKK1 is located between hiatus 3 and the combined hiatus 1 and 2. Consequently, the reliable bracketing ages for SKK 1 are 84.57 ± 0.76 ka at 6 cms dft (older layer), 83.32 ± 1.48 ka at 5.5 cms dft (same layer) and 56.7 ± 0.95 ka at 4 cms dft (younger layer) (Table 1, Fig. 3). The latter date corresponds to a warm MIS3 interval in the N. Hemisphere (GI-16.1). The DNA sample SKK3 was taken from the same spot as the u-series sample at 17.5 cm dft (Fig. 3) with an age of around 80.26 ± 1.87 ka and is capped by the u-series age 84.57 ± 0.76 ka at 6 cms dft (Table 1).

### Radiocarbon

A fragment of bone from the bottom of stalagmite SKK 16 5 was sent for AMS radiocarbon dating at the Research Laboratory for Archaeology and the History of Art, University of Oxford. The age of the bone is beyond the range of radiocarbon, giving it a minimum age of 50.200 BP uncalibrated (OxA-36539).

### Ancient DNA

All samples were aligned to the human reference genome (GRCh37/hg19) and damage patterns were assessed. Alignments to the human genome were either too short, <35 bp (base pairs), and aligned uniquely to the human genome or they were longer, >75 bp, and showed a low deamination rate, indicative of a high likelihood of contaminant modern human DNA (SI Fig. 2). As such, all primate sequences were excluded from further study due to potential for human contaminant DNA.

**Figure 5:**
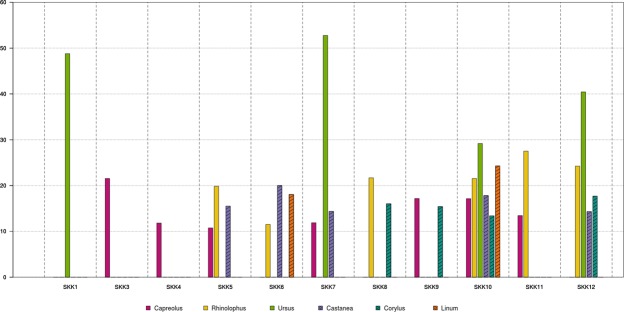
Deamination frequencies in the Solkota samples (graph made by DMF). The graph presents the average deamination frequencies at the 5′ and 3′ bases for the terminals ends. Only genera exceeding a 10% deamination threshold were accepted as ancient and are presented here. Solid bars represent mammalian genera, patterned bars represent plantae.

Analysis of the Melouri cave samples (MEL1–4) showed that the majority of aligned reads fell within the ranges of <35 bp, prone to misalignments, and >75 bp, with low deamination indicating potential contamination and low preservation of DNA of ancient origin (SI Table 2). The Melouri samples were therefore excluded from further analysis. In contrast, almost all Solkota cave samples (SKK1, 3–12) showed preservation of aDNA, most with multiple genera identifications (Fig. 5). In the case of SKK 2, screening prior to sequencing showed no discernible presence of DNA and this sample was therefore excluded from further analysis.

An initial global alignment to the Blastn database with MGmapper revealed 16 commonly occurring genera in the Solkota samples (SI Table 2). The reassessment for false positives (following the approach by Slon *et al*.^15^ and see method section below), positively identified 6 genera: *Capreolus* (roe deer), *Rhinolophus* (bat), *Ursus* (bear), *Castanea* (chestnut), *Corylus* (hazelnut), and *Linum* (flax) (Fig. 5 and SI Table 3). The combined number of uniquely aligned reads to the reference species of these genera (SI Tables 3 and 4) per speleothem section varied between 4541 (SKK12) and 72056 (SKK3), and the per-species damage patterns between 0 and 54% (Fig. 5, SI Table 3). The genus most frequently found was *Capreolus* (roe deer) which was positively identified in 7 of the 11 samples, followed by *Rhinolophus* (bat) in six, *Castanea* (chestnut) in five, *Ursus* (bear) and *Corylus* (hazelnut) in four, and *Linum* (flax) in only two (Fig. 5, SI Table 3). In SKK 10 we confirmed the presence of six ancient genera, the highest number among all Solkota samples. Interestingly, the samples from the bear bone embedded within the speleothems (SKK7, 10 and 12) also contained exogenous aDNA, including aDNA of other mammalia genera and plantae with damage patterns ranging from 11.87 to 27.50% (Fig. 5). The samples from the bear bone embedded in the speleothem matrix provided high numbers of aligned reads to Ursus (8465 for SKK7, but only 126/125 for SKK 10/12) and display a strong deamination pattern above 50% for SKK 7 and nearly 30% for SKK 10 and 12 (Fig. 5, SI Table 3). Together, the aligned reads for bear, the clear damage pattern, the minimum age of the bone and the zooarchaeological observations indicate that the speleothem embedded bone originates from cave bear. Sample SKK 1 also displayed a strong deamination pattern for bear reads, nearly 50%, here, however, no bear bone was present. Negative control analysis identified no ancient molecules aligned to any of the mentioned genomes (SI Table 3), indicating no cross-contamination between samples.

## Discussion and Conclusion

Growth phases of stalagmite SKK 16 3 can be linked to global climatic records. The speleothem’s rapid but intermittent growth around 84 ka coincides with climatic amelioration during Marine Isotope Stage (MIS) 5a, the Greenland Interstadial 21.1e (GI-21.1e)^37^. The resumption of growth at 56.7 ± 0.95 coincides with a warm interval in MIS3, Interstadial GS 16.1^37^. For both time periods, interstadial GI-21e and GI 16.1, no dates for human occupation in the region have been reported. However, this may be the result of limited dating of human occupation deposits beyond the range of radiocarbon in this region, many Middle Paleolithic sites still lack absolute dating (Bronze Cave, Sakaja and Ortvala^38^, Koudaro I, Undo^39^, Djruchula and Tsona^40^). Speleothem growth at Solkota Cave suggests episodic favourable climatic condition in the region during parts of MIS5 and MIS3, which could also have supported human occupation. However, climatic interpretations need to be further investigated with stable isotope data and can now also be coupled with environmental aDNA from the same stalagmite.

**Figure 6:**
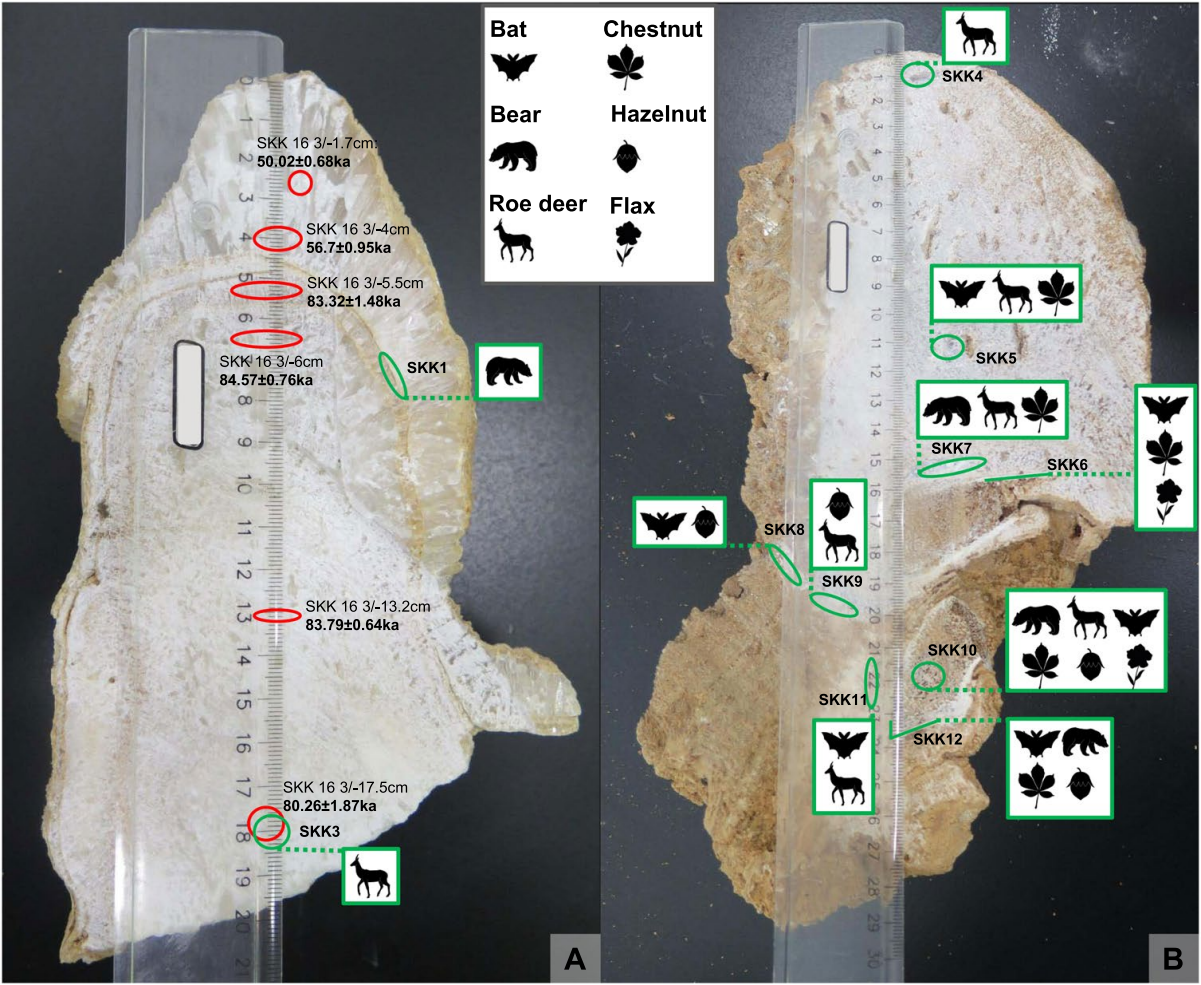
Genera identified by the aDNA analyses in stalagmites SKK 16 3 and 5 (photos taken by MCS). © MPI for Evolutionary Anthropology.

Our first metagenomic analyses presented here allowed the documentation of aDNA from inside the stalagmites with characteristic deamination damage to the DNA. We were able to identify the aDNA inside the stalagmites down to genera and to show the preservation of aDNA from mammals (bear, roe deer, horseshoe bat) and plants (chestnut, hazelnut, flax) from various layers inside the speleothem as well as from the incorporated bone (Fig. 6). The identified plants and large mammals indicate a generally forested environment. Similar landscape is also reconstructed from later Paleolithic sites of the area, such as Kotias Klde^41,42^ and Satsurblia^33,42^. Apart from sample SKK 2, all samples from stalagmites SKK 16 3 and 5 contained aDNA from one or more genera. SKK 1 and 3 from stalagmite SKK 16 3 gave each one genera confirmation, bear and roe deer respectively. For stalagmite SKK 16 5, the number of detected genera range from 1 to 6 per sample. Bone samples from this stalagmite (SKK7, 10, 12) exhibit a higher number of confirmed genera (4–6 per sample) than pure speleothem samples (SKK4, 5, 6, 8, 9, 11) (1–3 per sample). The preservation of aDNA with characteristic deamination damage in most of the studied samples show that both stalagmite and bone embedded in stalagmites are a promising medium for aDNA preservation. However, only for the aDNA from stalagmite SKK 16 3 absolutes ages could be inferred. SKK 3, containing roe deer, can be confidently assigned to be older than ~84 ka. The same layer that preserved the ancient bear DNA in SKK1, between hiatus 3 and the combined hiatus 1 and 2, was dated to 83.32 ± 1.48 ka with U-series. However, this layer is rather thin and it is possible that the aDNA sample contained dust from either hiatus event and the bracketing the U-series dates, 84.57 ± 0.76 ka (older layer) and 56.7 ± 0.95 ka (younger layer), provide a more reliable chronological frame. For stalagmite SKK 16 5 only a minimum age for the bone, >50.200 uncalibrated, could be deduced and the age of the stalagmite, which formed after the deposition of the bone, remains open.

The diverse occurrence of the aDNA with variability inside the stalagmites, between sampling contexts (pure speleothem versus bone embedded in the speleothem), between stalagmites from the same cave and from different caves of the same cave system, Solkota and Melouri Cave, open up the question of the formation history of the aDNA in this context. This formation history includes the DNA source, DNA adsorption, transport (agent), deposition and preservation of DNA inside the stalagmites. Zepeda Mendoza *et al*.^32^ noted in their analysis, that aDNA inside the studied popcorn speleothem contained aDNA originating from outside the cave as well as from different parts inside the cave. Similarly, in our study aDNA from cave dwelling genera (bear, bat) as well as non-cave dwelling genera (roe deer, hazelnut, chestnut and flax) are present. This mixture of allochthonous and autochthonous sources suggests also a mixture of depositional processes. A number of possible processes can be imagined. First, water is one possible transport agent, which infiltrates through soils above the cave through the epi-karst system into the cave, transporting plant, animal, bacterial, insect and fungal DNA. Another possible biogenic process is the direct contact of the organism, from which the DNA derived, with the speleothem, e.g. bears rubbing on speleothems, food remains (roe deer, nuts) adhering to the bear and being transported into the cave, bats and bear defecating and urinating. A final possible process is the gravitational transport of DNA adhering to sediment particles into the cave, as can easily be imagined with the steep entrance slope at Solkota cave. Clays, fine organic matter, silica grains and other minerals can all occur inside speleothems^32,43,44^. After deposition and adsorption of the DNA and formation of the speleothem, DNA preservation and integrity is promoted by the closed system of the stalagmite, making them a probably more reliable archive than sedimentary deposits and soils. In addition, precise dating of layers containing aDNA is possible. However, possible minor post-depositional migration of uranium in the lower part of speleothem SKK 16 5 (the lowermost age is out of stratigraphic order) opens up the question of the integrity of speleothem to post-depositional migration of aDNA. This being said, if DNA fragments are strongly adsorbed to sediment components, e.g. clay minerals^17–19^ deposited during the hiatus events within the speleothem, post-depositional migration by drip waters that percolate through the speleothem structure is less likely for the DNA than for water-soluble uranium.

The preservation of aDNA inside speleothems entails diverse prospects for archaeological and paleoenvironmental research. Paleoenvironmental speleothem records from cave sites are associated with contemporaneous archaeological, paleontological and paleobotanical records via correlating dates. The detection of mammalian and plant aDNA inside speleothems reveals a potentially direct link between these records. Furthermore, stalagmites can serve as an additional archive for old excavation, where all sediments, archaeological and paleontological remains have already been removed, or for sites where bone and overall organic preservation is poor.

## Materials and Methods

For sample extraction and to limit/control for sample contamination, speleothems were sawed only partially open with a rock saw, using deionized water for cooling, and were then broken open to reveal surfaces for sampling. After stratigraphic interpretation of the speleothems, samples were taken with a micro drill using layer-parallel elliptical sampling pits and including samples from the sawed and broken area (Figs 3 and 4). U-series samples were taken to constrain the age of calcite layers that contain aDNA and some were taken in tandem with the DNA samples, from adjacent/overlapping sampling locations. Sample size was 100–200 mg for U-series and 25–60 mg for DNA analysis. Samples for the latter were taken in a clean laboratory, using bleach to clean the surface of the speleothems and about 2 mm of the exposed inner speleothems surface was removed with the micro drill before sampling to limited contamination. Samples for DNA sequencing include samples from the speleothem as well as samples from the bone inside speleothem SKK 16 5, cortical and trabecular bone (Fig. 4).

### U-series

Methods for U-series dating methods were similar to those described by Fankhauser *et al*.^44^. Briefly, sample powders were weighed and spiked with a mixed ^233^U/^236^U/^229^Th tracer. Following dissolution and spike equilibration, separation of uranium and thorium was completed by anion exchange chromatography. All measurements were carried out using a ThermoFisher Neptune® high-resolution inductively coupled plasma mass spectrometer with an Aridus® desolvation nebuliser at the School of Earth Sciences, University College Dublin. ^238^U/^236^U and ^233^U/^236^U ratios were measured using three Faraday collectors, while the ^234^U ion beam was measured in a secondary electron multiplier (SEM). Calibration of the SEM relative to the Faraday detectors was achieved by sample-standard bracketing, using the certified ^235^U/^238^U ratio of the IRMM-3184 standard. Mass-fractionation corrections for uranium were applied based on the certified ^233^U/^236^U ratio of the mixed spike. The minor isotopes of thorium (^230^Th and ^229^Th) were measured using the SEM, whilst two Faraday collectors were used to simultaneously measure the much larger ^232^Th ion beam. A standard-sample bracketing method using the IRMM-3184^44^ standard a uranium standard was applied for the thorium mass fractionation correction and for the SEM/Faraday yield calibration.

As discussed in the results, several sub-samples from the speleothem contained significant amounts of non-carbonate ‘detrital’ thorium as evidenced by high ^232^Th concentrations and low ^230^Th/^232^Th ratios (Table 1). This necessitated corrections for inherited non-radiogenic ^230^Th. In the literature this correction is often achieved by simply assuming that the ^230^Th/^232^Th ratio of the inherited (non-carbonate) fraction is equivalent to a typical upper crustal ^238^U/^232^Th activity ratio of 0.8 ± 0.4^45^. In this study, in order to reduce the dating uncertainties associated with the detrital correction we measured the U-series isotope ratios in clay-rich samples from the cave to constrain the actual non-carbonate (silicate clay mineral) ^230^Th/^232^Th ratio.

### Ancient DNA

DNA samples were extracted and prepared within a clean room environment at a dedicated ancient DNA laboratory at University College Dublin (UCD), Ireland. Unilateral air-flow hoods, tyvek suits, hair nets, face masks and non-powdered gloves were used to limit contamination. Upon amplification further steps were performed in a modern laboratory environment. DNA extraction was undertaken according to the method outlined by Collin *et al*.^46,47^ (Collin manuscript in preparation). This protocol, developed for the extraction of aDNA from anthropogenic sediments, reduces the action of potentially damaging geopolymers on DNA by chemical inhibition and increases the range as well as quantity of isolated DNA fragments thereby reducing dependency on DNA capture techniques for exploratory samples. The extraction protocol consisted of samples being placed into Matrix E lysing tubes (MP-BIO-116914050) and submerging them in 1 mL of extraction buffer up to a final concentration of 0.45 M EDTA, 0.02 M TrisHCL (pH 8.0), 0.025% SDS, 0.5 mg/mL Proteinase K and dH2O. Samples were incubated at 39 °C overnight using an Eppendorf Thermomixer® C with a rotational speed of 1600rpm to ensure maximal bead movement. Supernatant was collected and cleaned following Dabney *et al*.^48^ and DNA libraries were prepared following Meyer and Kircher^49^. Negative controls were included at all stages and pooled to investigate the presence of damaged reads indicative of cross-contamination during DNA extraction and library preparation.

DNA samples were amplified using a universal Illumina primer and Polymerase chain reaction (PCR) following Gamba *et al*.^50^ and were repeated 15 times following Collin *et al*.^46,47^. Assessment of PCR reaction concentrations were performed on an Agilent 2100 Bioanalyser following instructions of the manufacturer. Based on these concentrations samples were pooled into a 4 nM working solution and sequenced on an Illumina NextSeq. 500/550 using the high output v2 (75 cycle) reagent kit at UCD Conway Institute of Biomolecular and Biomedical Research. Genera were initially identified by cross referencing raw sequencing data with the National Centre for Biotechnology (NCBI) genomic database using Basic Local Alignment Search Tool (BLAST)^51–53^ at an evalue of 1e-05 and MGmapper^54^ with a 0.8 fraction of matches + mismatches and minimum alignment score of 25^47^. In order to reduce the chances of false positive identifications of taxa at the family or genus level we followed a similar approach to that of Slon *et al*.^15^. After the initial alignment using Blastn, an offline nucleotide BLAST + database was generated with the genomic sequences of the 16 main eukaryote species detected (12 animals and 4 plants, SI Table 2) with “makeblastdb” (genome versions for each species presented in SI Table 4). Each sample’s trimmed reads were aligned to this database using default “blastn” parameters and the resulting output data was imported into MEGAN Community Edition v.6.2.13^55^. For the last common ancestor (LCA) parameters we used a minimum bitscore of 35 within the top 10% of the best alignments, minimum support count of 2, and the default “naive” LCA algorithm^15^. A minimum 1% of the total assigned reads was necessary to accept a taxa to be present following Slon *et al*.^15^ and were then used for downstream analysis (SI Table 3). The sets of reads assigned to each species were extracted into independent files and then aligned to the correspondent genome for authentication. We used BWA v.0.7.5a-r405 “aln”^56^ with permissive parameters (−o 2 −n 0.01) and disabled seed (-l 1000), and then the aligned reads were filtered for a minimum quality of 25, sorted, duplicates removed, and indexed using samtools v.1.3.1^57^. Using mapDamage2^57^ we investigated and quantified the presence of C to T substitutions on the 5’ end and G to A on the 3’ end of the sequences, and used a minimum value of 10% on both sides for a taxon to be identified as ancient^6,16^. Average read lengths were calculated using Genome Analysis Toolkit’s “ReadLengthDistribution” (see SI Fig. 3 for an averaged deamination length plot)^58^.

## Supplementary information

Stahlschmidt et al SI Text 1 Figure 1 to 3 Table 1

SI Tables 2-4 revised
