# Supplementary material for: Ancient Mammalian and Plant DNA from Late Quaternary Stalagmite Layers at Solkota Cave, Georgia
Source: Sci Rep. 2019 Apr 29;9:6628. doi: 10.1038/s41598-019-43147-0 (PMC6488622; doi:10.1038/s41598-019-43147-0)
Supplement: Supplementary file 1 — Stahlschmidt et al SI Text 1 Figure 1 to 3 Table 1 [file 41598_2019_43147_MOESM1_ESM.docx]

**SUPPLEMENTARY MATERIAL**

**Ancient Mammalian and Plant DNA from Late Quaternary Stalagmite Layers at Solkota Cave, Georgia**

M. C. Stahlschmidt^1,2^*, T. C. Collin^3^, D. M. Fernandes^4,5^, G. Bar-Oz^6^, A. Belfer-Cohen^7^, Z. Gao^8^, N. Jakeli^9^, Z. Matskevich^10^, T. Meshveliani^9^, J. K. Pritchard^8,11,12^, F. McDermott^13^, R. Pinhasi^4^*

^1^ Department of Human Evolution, Max-Planck-Institute for Evolutionary Anthropology, Leipzig, Germany.

^2^ School of Archaeology, University College Dublin, Dublin, Ireland.

^3^ School of Medicine, University College Dublin, Dublin, Ireland.

^4^ Department of Evolutionary Anthropology, University of Vienna, Vienna, Austria.

^5^ CIAS, Department of Life Sciences, University of Coimbra, Coimbra, Portugal.

^6^ Zinman Institute of Archaeology, University of Haifa, Haifa, Israel.

^7^ Institute of Archaeology, The Hebrew University of Jerusalem, Jerusalem, Israel.

^8^ Department of Genetics, Stanford University, Stanford, U.S.A.

^9^ Department of Prehistory, Georgian State Museum, Tbilisi, Georgia.

^10^ Israel Antiquities Authority, Jerusalem, Israel.

^11^ Departments of Biology, Stanford University, Stanford, U.S.A.

^12^ Howard Hughes Medical Institute, Stanford University, Stanford, U.S.A.

^13^ School of Earth Sciences, University College Dublin, Dublin, Ireland.

*corresponding authors: [mareike_stahlschmidt@eva.mpg.de](mailto:mareike_stahlschmidt@eva.mpg.de), [ron.pinhasi@univie.ac.at](file:///D:\Users\mareike_stahlschmidt\Documents\Projects%20SEE%20SHARED%20FILES\Speleothems%20and%20aDNA\Georgia%20Speleothems\Papers\Scientific%20Reports\for%20submission%20Science%20Advances\ron.pinhasi@univie.ac.at)

**SI Text 1**

Melouri Cave is in walking distance of Solkota and similarly located in collapsed sinkhole (SI Fig 1a). Melouri cave has a length of 5.3 km, a maximum width of 30m and a maximum height of 62 m (data from a sign in front of the cave). Little light penetrates into the cave and it is rich in speleothems (stalagmites, stalactites, flowstones and curtains). We collected five speleothem samples in total (3 stalagmites, 1 bedrock and 1 flow stone sample). No archaeological remains were observed inside the cave. We opened and sampled one stalagmite, MEL 16 5 (SI Fig 1b). Four samples were collected for aDNA analysis with a microdrill after removing 2 mm of the surface (see sampling and method description in the main text). The analyses of sequence data from MEL 16 5 revealed potential contamination and low preservation of DNA (see main text and SI Table 3).

**SI Figure 1. Melouri Cave** (photo by MCS)**.**


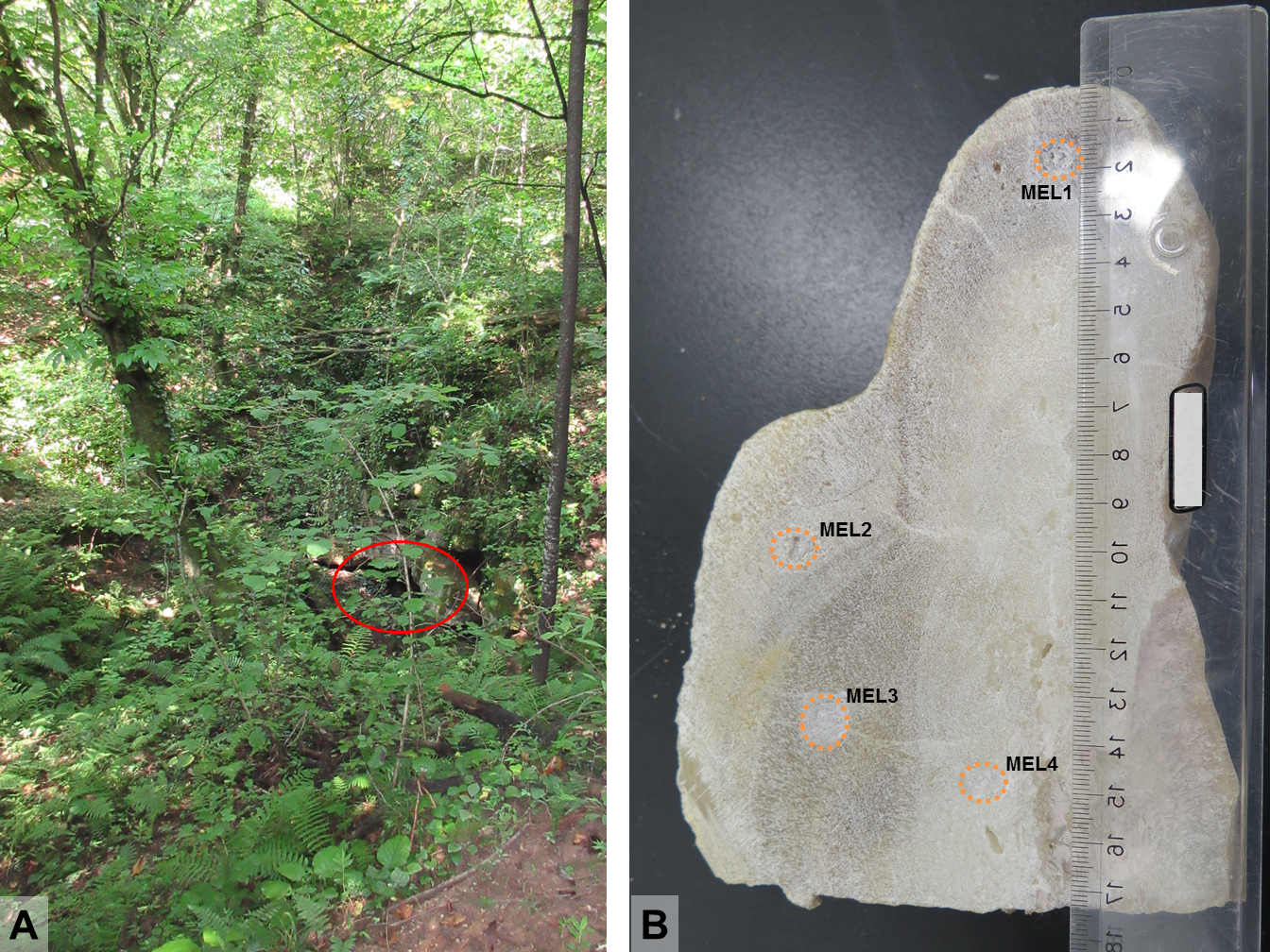


(**A**) Narrow entrance (red circle) to Melouri Cave in a karstic depression. (**B**) Stalagmite MEL 16 5 cut open and sampled for aDNA analysis (yellow dotted line, unsuccessful). Note the clear layering of the stalagmite and differential color indicating different amounts of detrital clay. Sampling was aimed at testing aDNA preservation in these different contexts. However, no aDNA was detected.

**SI Figure 2. Alignment distribution** (graph by TCC)**.**

Sample MEL2 single-end read length distribution aligned to the human genome. The majority of the alignments were either below 35bp or above 75bp. The reads below 35 bp are subject to misalignments and as such are too short for confirmation of origin and the alignments above 75 bp exhibited a low deamination rate, indicating contamination of the sample.

**SI Figure 3. Deamination frequency** (graph by TCC)**.**


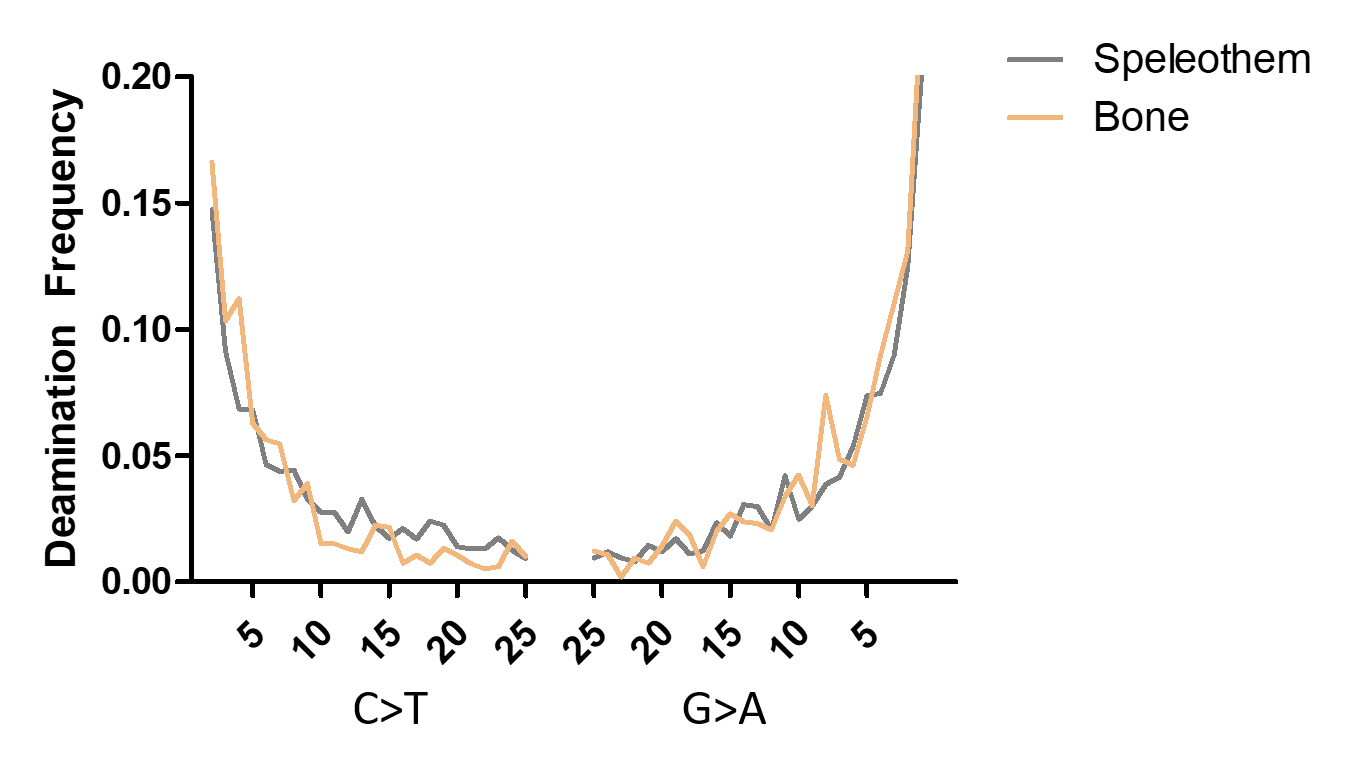
 Averaged deamination frequency for all speleothem and speleothem embedded bone samples showing that deamination increases across all 25bp on the C>T and G>A terminal ends, demonstrating that the DNA is ancient.

**SI Table 1. Unreliable U-series dates.**

| **Sample** | **^238^U ppb** | **(^230^Th/^238^U)** | **(^234^U/^238^U)** | **(^230^Th/^232^Th)** | **^232^Th ppb** | **Age ka uncorrected** | **Age ka corrected** |
| --- | --- | --- | --- | --- | --- | --- | --- |
| SKK16 3/10 | 44.227±0.007 | 0.8181±0.0012 | 1.1180±0.0014 | 2.604±0.004 | 42.456±0.0094 | 138.42±0.78 | 67.37±^14.65^ _12.94_ |
| SKK16 3/6.5 | 54.518±0.009 | 0.9797±0.0013 | 1.1152±0.0015 | 2.127±0.003 | 76.745±0.019 | 212.06±^1.77^ _1.74_ | 71.68±^25.50^ _20.75_ |
| SKK16 3/5 | 72.656±0.008 | 0.7292±0.0084 | 1.1263±0.0009 | 3.214±0.014 | 50.37±0.28 | 110.70±^2.33^ _2.28_ | 65.59±^9.69^ _8.91_ |
| SKK16 5/1.5 | 34.881±0.007 | 0.9652±0.0019 | 1.0827±0.0015 | 0.959±0.002 | 107.305±0.036 | 226.34±^2.64^_2.57_ | Unable to calculated |
| SKK16 5/11.5 | 31.033±0.004 | 0.6250±0.0018 | 1.0905±0.0011 | 0.962±0.003 | 61.586±0.021 | 91.51±^0.55^_0.55_ | Unable to calculated |
| SKK16 5/15 | 166.754±0.018 | 0.8231±0.0025 | 1.1272±0.0006 | 1.827±0.005 | 229.613±0.177 | 137.50±^0.95^_0.94_ | 46.69±^21.21^_17.85_ |
| SKK16 5/20 | 56.966±0.007 | 1.7574±0.0119 | 1.0718±0.0008 | 1.031±0.005 | 296.78±0.66 | 800.00±^0.00^_0.00_ | Unable to calculated |

U-series data for SKK 16 3 and 5 with unreliable age calculation. Parentheses denote activity ratios. Dates reported in this table are strongly affected by detrital correction and reliable dates could not be calculated. The following decay constants were used: 230Th: 9.1577E-6, 232Th: 4.9475E-11, 234U: 2.826E-6, 238UE 1.551E-10. The final column on the right hand side shows the ages calculated after correction for detrital thorium using a measured (230Th/232Th) value of 0.95±0.1 for the detrital end-member.
